# Allele-specific Characterization of Alanine: Glyoxylate Aminotransferase Variants Associated with Primary Hyperoxaluria

**DOI:** 10.1371/journal.pone.0094338

**Published:** 2014-04-09

**Authors:** Melissa D. Lage, Adrianne M. C. Pittman, Alessandro Roncador, Barbara Cellini, Chandra L. Tucker

**Affiliations:** 1 Department of Biology, Duke University, Durham, NC, USA; 2 Department of Life Sciences and Reproduction, Section of Biological Chemistry, University of Verona, Verona, Italy; 3 Department of Pharmacology, University of Colorado Denver School of Medicine, Aurora, CO, USA; Universidad de Granada, Spain

## Abstract

Primary Hyperoxaluria Type 1 (PH1) is a rare autosomal recessive kidney stone disease caused by deficiency of the peroxisomal enzyme alanine: glyoxylate aminotransferase (AGT), which is involved in glyoxylate detoxification. Over 75 different missense mutations in AGT have been found associated with PH1. While some of the mutations have been found to affect enzyme activity, stability, and/or localization, approximately half of these mutations are completely uncharacterized. In this study, we sought to systematically characterize AGT missense mutations associated with PH1. To facilitate analysis, we used two high-throughput yeast-based assays: one that assesses AGT specific activity, and one that assesses protein stability. Approximately 30% of PH1-associated missense mutations are found in conjunction with a minor allele polymorphic variant, which can interact to elicit complex effects on protein stability and trafficking. To better understand this allele interaction, we functionally characterized each of 34 mutants on both the major (wild-type) and minor allele backgrounds, identifying mutations that synergize with the minor allele. We classify these mutants into four distinct categories depending on activity/stability results in the different alleles. Twelve mutants were found to display reduced activity in combination with the minor allele, compared with the major allele background. When mapped on the AGT dimer structure, these mutants reveal localized regions of the protein that appear particularly sensitive to interactions with the minor allele variant. While the majority of the deleterious effects on activity in the minor allele can be attributed to synergistic interaction affecting protein stability, we identify one mutation, E274D, that appears to specifically affect activity when in combination with the minor allele.

## Introduction

Primary Hyperoxaluria Type 1 (PH1) is a severe autosomal recessive kidney stone disease, caused by loss or dysfunction of the enzyme alanine: glyoxylate aminotransferase (AGT). AGT is a hepatic peroxisomal enzyme involved in glyoxylate metabolism that detoxifies glyoxylate in the peroxisomes of cells by converting it to glycine [1]. If not degraded, excess glyoxylate results in buildup of oxalate, which is deposited in the kidneys in the form of calcium oxalate, resulting in nephrocalcinosis, urolithiasis, and renal failure [1].

The AGT enzyme exists in two polymorphic variants, termed the major and minor alleles (herein depicted ‘AGTma’ and ‘AGTmi’). The minor allele variant is found in approximately 15–20% of European and North American populations and results in two amino acid changes, a Pro11Leu substitution at the N-terminus of the protein, and a Ile340Met substitution at the C-terminus [2]. Prior studies *in vitro* and *in vivo* have established that AGTmi shows both reduced activity and stability [3]–[7]. These properties have been attributed almost entirely to the P11L substitution [3], [5], [6], [8], [9], which in addition to affecting stability and activity also generates a weak mitochondrial targeting signal at the N-terminus of the protein [2], [10].

While the minor allele P11L substitution is not itself disease-causing, Leu11 appears to interact synergistically with other mutations to cause PH1. These interactions result in a variety of phenotypic effects. In some cases, the addition of a second mutation appears to have primarily a destabilizing role, acting together with Leu11 to generate a severely destabilized and thus non-functional enzyme. In other cases, the mutations have more complex effects on protein trafficking [6], [11]. For example, a G170R mutation found on the minor allele is observed in ∼30% of PH1 patients [2]. This allele has been found to show only a minor (∼50%) [3], [5], [7] to no [9], [12] defect in catalytic activity in purified protein or whole cell enzymatic assays. However in patients and mammalian cell culture, a majority of the AGTmiG170R mutant is mislocalized to the mitochondria rather than the peroxisome [13], [14]. The P11L substitution generates a weak mitochondrial targeting signal that by itself does not appear to result in significant mistargeting of AGTmi, as the dimer form of the protein appears to restrict or hide the N-terminus, thus preventing contact with the mitochondrial targeting machinery [2], [14], [15]. However, the G170R substitution is thought to promote protein aggregation and unfolding that may maintain the protein for a longer time in an unfolded or partially folded state where it shows enhanced interaction with cytosolic chaperones [1], [3], [9], [12], [14]. In this unfolded/partially folded state, the protein may be more easily targeted for mitochondrial, rather than peroxisomal, trafficking.

In addition to G170R, over 25 other disease-associated mutations have been found only in combination with the minor allele [11]. While some of these alleles have been characterized in depth biochemically, a number have not been characterized at all. In previous work, we developed yeast-based assays to characterize AGT specific activity and stability. The activity assay is based on complementation of a yeast alanine: glyoxylate aminotransferase deficiency with the human enzyme [7]. The stability assay (IDESA) couples growth of yeast to activity of a hybrid dihydrofolate reductase (DHFR)-AGT construct, wherein mutations that destabilize AGT also destabilize DHFR and reduce growth of yeast dependent on DHFR activity [16]. In this study, we used these two assays to characterize mutations in AGT found in PH1 patients, determining effects of these mutations on protein specific activity and stability. As controls, we also included some alleles that have been extensively characterized, including W108R, G161C, and G161S [4], [16]–[19]. To better understand how specific mutations interact synergistically with the minor allele polymorphism, we examined the majority of the mutations on both alleles–minor and major. Here we describe the results of this study, in which we systematically characterize activity and stability of 34 variants on major and minor alleles. By clustering mutant phenotypes based on how the mutations affect activity and stability in combination with the major or minor alleles, we classify the mutants into 4 subclasses that allow better understanding of the subtypes of molecular defects associated with PH1.

## Materials and Methods

### 

#### Strains and Constructs

Strains used were TH5 (*MATa leu2-3,112 trp1 ura3-52 dfr1::URA3 tup1*) and YCG-F^r^ (*MATa ura3-1 trp1-1 ade2-1 his3-11,-15 leu2-3,-112 can1-100 shm1::HIS3 shm2::LEU2 gly1::URA3 AGX1::kanMX4*). For activity assays, mutations were generated by PCR and cloned via homologous recombination into p416GPD-AGTma or p416GPD-AGTmi. For stability assays, AGT mutants from activity assays were cloned into pTB3-dhAGT [16].

#### Yeast-based assays

The yeast AGT activity assay was carried out using YCG-F^r^ as described previously [7]. The stability assay was carried out using the TH5 strain as described [16], growing yeast at 30°C. Growth was quantified by measuring the turbidity of the yeast cultures at OD_600_ using a SpectraMax 190 plate reader (Molecular Devices) after 68–78 hours (activity assay) or 40–48 hours (stability assay). Assays were carried out at least three independent times, and average and standard error reported for each mutant. For graphs and analysis, mutants expressed on the AGTma background were normalized to growth of AGTma, while mutants on the AGTmi background were normalized to growth of AGTmi.

#### Bacterial protein expression and characterization

AGTmi^E274D^ was cloned into bacterial expression vector pET30a and purified using previously described methods [7]. Assays of AGT activity were carried out as previously described [7]. Preparation of apo-AGT was carried out as described [20]. Absorption measurements were made with a Jasco V-550 spectrophotometer with a 1 cm path length quartz cuvette at a protein concentration of 10 μM. Intrinsic fluorescence emission spectra were recorded on a Jasco FP-750 spectrofluorometer equipped with a thermostatically controlled cell holder. Spectra of the blanks, i.e. samples containing all components except protein, were taken immediately before the measurement of the sample containing AGT.

The molecular dimensions of AGTmi^E274D^ at 10 μM concentration in the presence of 100 μM PLP were determined by size-exclusion chromatography. The mixture was loaded on a custom packed Sephacryl S-300 10/600 column equilibrated and run with 100 mM potassium phosphate buffer pH 7.4 on an Akta FPLC system (GE Healthcare). The injection volume was 500 μl at a flow rate of 0.4 ml/min with detection at 280 nm. The software Unicorn 5.01 (GE Healthcare) was used to calculate the elution volume and the area of each peak. The apparent hydrodynamic radius of the eluting species was calculated by comparing its elution volume to that of a set of molecular weight standards under the same experimental conditions.

The amount of PLP bound to AGTmi and AGTmi^E274D^ upon incubation with excess exogenous PLP and dialysis to remove unbound coenzyme was determined by HPLC analysis on a 5-μm Supelcosyl C18 column (250×4.6 mm) connected to a Jasco PU2080 HPLC control system. The eluent was 50 mM potassium phosphate buffer, pH 2.35, at a flow rate of 1 ml/min. An UV 275 plus detector set at 295 nm was employed. The enzyme mixture was denatured by adding trichloroacetic acid to a final concentration of 10% and then centrifuged to remove the precipitated protein.

#### Mammalian protein expression and characterization

AGTmi wild-type or mutant cDNAs were cloned into pCDNA3 (Life Technologies) at EcoRI and BamHI sites. Constructs were transiently transfected into COS-7 cells using Lipofectamine2000 (Life Technologies) according to the manufacturer’s protocols. For immunofluorescence staining, cells were fixed on coverslips with 4% paraformaldehyde, permeabilized in 0.2% Triton-X-100/5% normal goat serum in PBS, and incubated with either an anti-PMP70 primary antibody (Sigma) to label peroxisomes, or MitoTracker (Molecular Probes) to label mitochondria. Images were collected using a Zeiss LSM 510 confocal microscope, provided by the University of Colorado School of Medicine Advanced Light Microscopy Core facility. For western blotting, cells were lysed by boiling in 2x Laemmli Sample Buffer and run on an SDS-PAGE gel. Proteins were transferred to a nitrocellulose membrane and blotted with an anti-AGT antibody (kindly provided by Chris Danpure).

## Results

Over 75 different missense mutations have been linked to PH1 in patients. We chose 34 of these mutations (Table 1) to examine in a yeast cell based activity assay [7] and stability assay [16]. The activity assay relies on complementation of the yeast alanine: glyoxylate aminotransferase (AGX1) with the human enzyme. In yeast, deletion of the alanine: glyoxylate aminotransferase enzyme is not lethal, but yeast lacking this protein in a *shm1*, *shm2*, *gly1* background are unable to grow on media containing ethanol [21]. Human AGT can complement the yeast deficiency, allowing growth on ethanol, and mutant forms of AGT that have reduced activity *in vivo* and *in vitro* show reduced growth in this assay [7]. The stability assay relies on a dihydrofolate reductase (DHFR) fusion that complements a DHFR deficiency in yeast. A target protein of interest is inserted internally in DHFR, linking stability of the target protein with that of DHFR. As DHFR is essential, growth of yeast correlates with DHFR activity, such that mutations that reduce stability decrease growth of yeast [16].

**Table 1:** List of PH1-associated mutations tested.

Mutation	Allele	Prior Activity Analysis	Prior Stability Analysis
**Group I**			
L25R	?		
G109V	Mi		
R111Q	?		
G161R	Ma	ma[5], [18]	mi[16]/ma[18]
M195R	Mi		
D201E	Ma		
L269P	Ma		
L284P	Mi		
L298P	Mi		
L359P	Ma		
L384P	Ma		
**Group II**			
I56N	Ma Mi		
E95K	Mi	mi[11]	
G161C	Mi	mi[18], [19], [24]/ma[24]	mi[18], [19]
G161S	Mi	mi[18], [19], [24]/ma[24]	mi[18], [19]
L166P	Mi	mi/ma[24]	
M195L	Mi		
D243H	?		
C253R	Mi	mi/ma[16], [24]	
P319L	?	mi[9]/ma[19]	mi[9]/ma[19]
A368T	Mi	mi[9]	mi[9]
**Group III**			
D129H	Ma		
L153V	Ma		
I279M	?		
Q282R	Ma		
S287T	?		
**Group IV**			
R36C	Mi	mi[16], [24]/ma[24]	mi[16]
G47R	Mi	mi[16]	mi[16]
G82R	Mi	mi[16]	mi[16]
H83R	Mi	mi[9], [11], [16]	mi[9], [16]
W108R	Mi	mi[4],[16],[19]	mi[16], [19]
H261Q	?	mi[16]	mi[16]
E274D	Mi	mi[16]	mi[16]
S275R	Ma Mi	mi[16]	mi[16]

A number of AGT mutations from PH1 patients are found only in association with a minor allele polymorphic variant, AGTmi. We examined the interaction between each of the 34 mutants and AGTmi and AGTma, characterizing the effect of the mutation on activity and stability of each polymorphic allele. We then clustered the mutants according to four different classifications (noted in Table 1) reflecting how the mutations affect protein activity and stability: mutations defective in activity and stability but not synergistic with the minor allele (Group I); mutations defective in activity and stability, synergistic with the minor allele (Group II); mutations showing no defects (Group III); and mutations showing defects in activity but not stability (Group IV).

**Figure 1:**
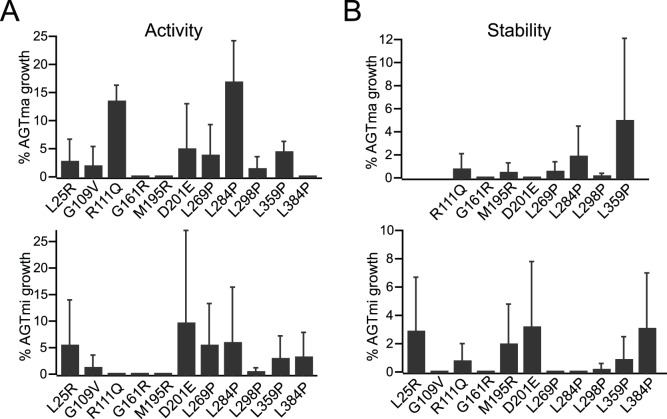
Group I: mutants defective in activity/stability in both alleles. Indicated mutations in AGTma (top) or AGTmi (bottom) were tested for effects on activity (**A**) or stability (**B**). Growth of mutants was normalized to % growth of wild-type. Average and standard error of at least three independent assays is presented.

The largest number of mutants (‘Group I’, 11 total) showed defects in activity in both the major and minor alleles, defects in stability in the minor allele, and for 8 of 11 mutants analyzed, defects in stability in the major allele (Fig. 1). Of this set of 11 mutants, ten were previously uncharacterized (Table 1). Fig. 1A shows activity of each allele in the context of the wild-type sequence (AGTma, top) or with the additional P11L, I340M mutations (AGTmi, bottom), while Fig. 1B shows the effects of each mutation on stability. With the exception of L25R, G109V, and L384P, which we did not analyze due to cloning issues, mutants were analyzed for stability effects in both alleles. We found that all of these variants showed <15% wild-type activity and stability in both AGTma and AGTmi. The one variant for which activity had previously been determined, AGTma G161R (0.3–6.2% activity compared with AGTma) [5], [18] showed similar activity in our analysis. Based on our results, we speculate that Group I mutations cause loss of activity due to global protein destabilization. Four of these mutations consist of Leu to Pro substitutions in α-helical domains. Such substitutions are often very deleterious, as the insertion of the proline distorts the secondary structure. In this set of mutants, we found that generally both AGTma and AGTmi are equally destabilized and show equivalent loss of activity. Although four mutants in this category were found only in patients in association with AGTmi, these results showing defects in activity when combined with AGTma suggest that these mutations would likely cause disease if found in conjunction with the AGTma allele as well.

**Figure 2:**
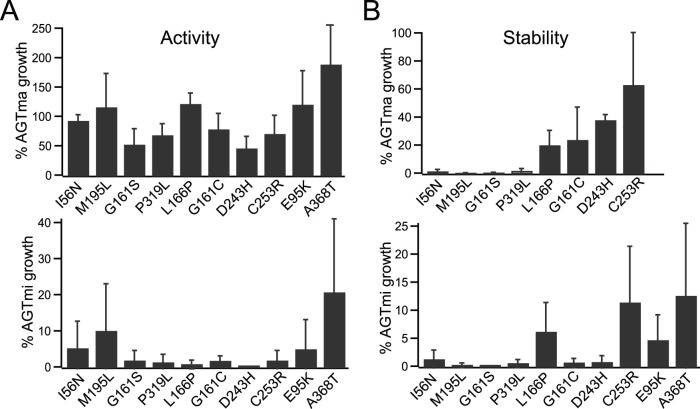
Group II: mutants defective in activity/stability, greater effects in AGTmi allele. Indicated mutations in AGTma (top) or AGTmi (bottom) were tested for effects on activity (**A**) or stability (**B**). Growth of mutants was normalized to % growth of wild-type AGTma (top) or AGTmi (bottom). Average and standard error of at least three independent assays is presented.

A second group of mutants (‘Group II’, 10 total) also showed defects in activity and stability in AGTmi, but exhibited good activity in the AGTma background (Fig. 2). Eight of these mutants were originally found on the AGTmi background in patients (with one, I56N, also found associated with AGTma) [11]. For two mutants (D243H and P319L) the patient allele has not been identified [22], [23]. Three of these variants (I56N, M195L, and D243H) are completely uncharacterized, while the other seven have been characterized for effects on activity (Table 1) [9], [24]. Four (G161S, G161C, P319L, and A368T) have been analyzed for stability effects *in vitro* and in a cellular model system [9], [18]. *In vitro* measurements of activity have varied, depending on differences in expression and purification conditions: characterization of AGTmi G161S, G161C, L166P, and C253R from one group showed <1% AGTmi activity [24], while a separate analysis revealed a more mild defect for AGTmi G161C and G161S (catalytic efficiency equal to about 30% and of 60% of AGTmi, respectively), but found that only ∼10% of the purified protein was soluble [19]. Further analysis of the G161C and G161S mutants in a cell culture model confirmed low steady-state protein levels in comparison to AGTmi, and showed that this is due to the presence of significant amounts of aggregates within cells derived from the apo-form of the variants [18]. In our *in vivo* assay, all ten Group II variants showed poor growth in the activity assay (0–20% of wild-type) and stability assay (0–10% of wild-type) in AGTmi, but retained near wild-type activity (50–100% of wild-type) in AGTma. In the previously mentioned *in vitro* analysis of four mutants (G161S, G161C, L166P, and C253R), significantly greater activity in AGTma compared with AGTmi was also observed [24]. In the AGTmi background, Group II mutants appear to have defects in stability that correlate with a defect in activity, similar to Group I. However, unlike Group I, the mutations appear to interact synergistically with AGTmi, as activity is not significantly impaired in the AGTma background. Stability with AGTma was variable in this group, with one variant, C253R, showing ∼60% of wild-type stability, but all others showing much less. While the activity assay would suggest that all proteins in this group maintain a native, folded conformation (a requirement for activity) in AGTma, the stability assay, which is a more stringent measure of stability involving insertion of each protein into the reporter DHFR, suggests that the stability of some of these mutants (I56N, M195L, G161S, P319L, L166P, and G161C) is not equivalent to that adopted by wild-type AGTma. In agreement with this, a strongly reduced thermal stability of mi-G161S and mi-G161C in the purified form, and a strongly reduced half-life and an increased propensity to aggregation of the variants expressed in eukaryotic cells have been reported [18], [19].

**Figure 3:**
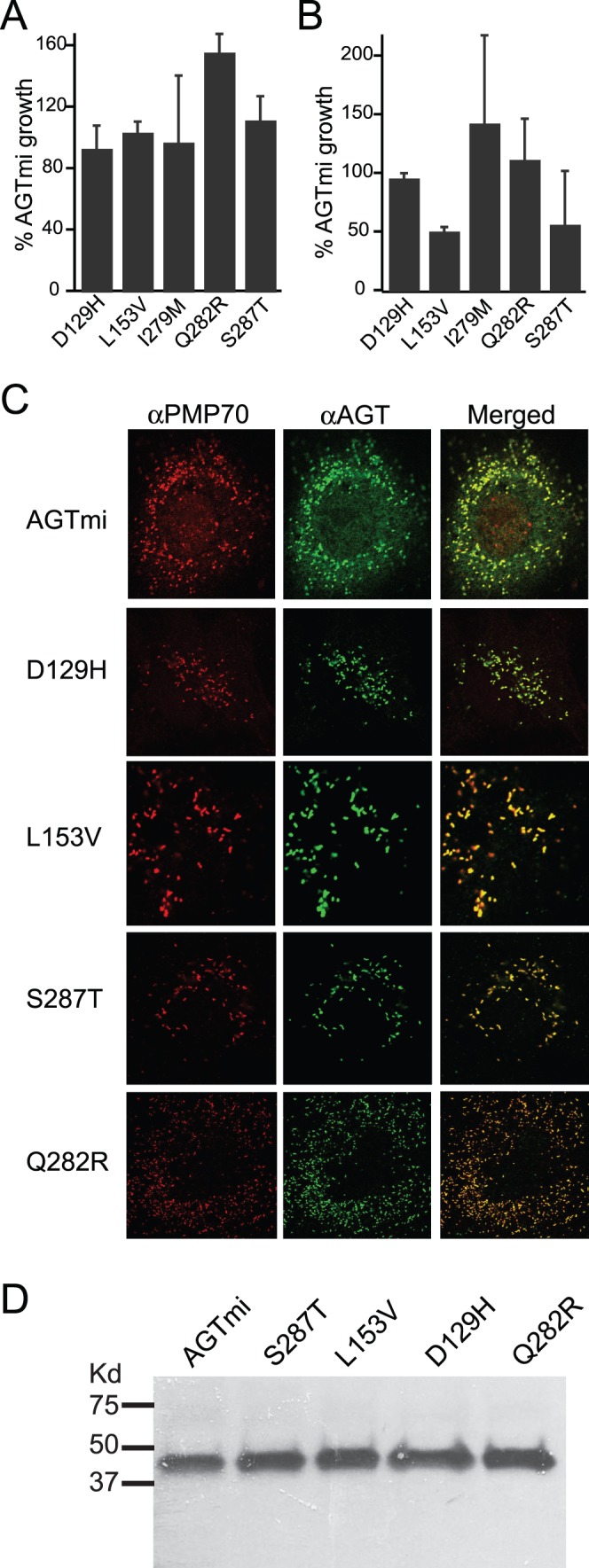
Group III: mutants that behave similar to wild-type. Indicated mutations in AGTma (top) or AGTmi (bottom) were tested for effects on activity (**A**) or stability (**B**). Growth of mutants was normalized to % growth of wild-type. Average and standard error of at least three independent assays is presented. (**C**) Localization of mutants (in AGTmi background) during heterologous expression in COS-7 cells. All mutants tested localized normally to the peroxisome (labeled with an anti-PMP70 marker). (**D**) Western blotting of mutants expressed in COS-7 cells using an anti-AGT antibody.

Group III consists of five mutants that have not been previously characterized, which showed similar activity and stability (within 50–100%) as wild-type AGT. Shown in Figure 3 are the activity (Fig. 3A) and stability (Fig. 3B) measurements of these mutants in AGTmi. As we did not see a substantial deficit in the AGTmi background, which is less stable and has less activity than AGTma, we did not generate these mutants in combination with AGTma. Overall, these variants showed near wild-type growth in both assays, with the exception of L153V and S287T that showed ∼50% reduction in growth in the stability assay. Althought we cannot exclude that the reduction in stability seen with L153V and S287T may contribute to the disease phenotype, these mutants are are clearly in a different class than the other classes of mutants analyzed, which generally showed severe defects.

We speculated that these proteins could show near wild-type activity and stability in the heterologous yeast assays, but the mutations could affect peroxisomal trafficking in mammalian cells. To examine this, we expressed four of these variants (D129H, L153V, S287T, Q282R) in the context of the minor allele in COS-7 cells and examined localization (Fig. 3C) and expression (Fig. 3D). All constructs showed wild-type peroxisomal localization, as assessed by colocalization with PMP-70, a peroxisomal marker (Fig. 3C), and by absence of colocalization with Mitotracker, which labels mitochondria (data not shown). Steady-state expression of variants visualized by western blotting showed no differences compared with AGTmi expression levels. While the mutants were analyzed in the AGTmi background and we cannot exclude the possibility that they could show effects in AGTma or could have more complex effects in hepatocytes, based on their normal expression and localization in COS-7 cells and wild-type activity in the yeast growth assay we postulate that they could represent amino acid changes that, although found in patients, are not deleterious or causative of disease.

**Figure 4:**
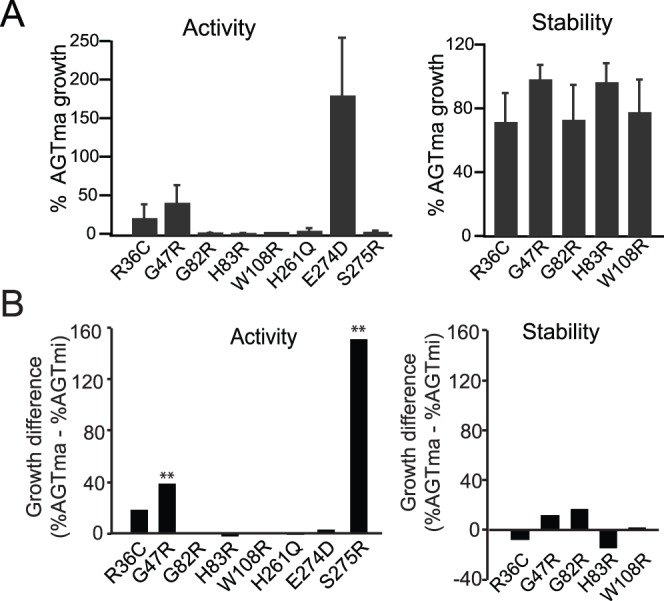
Group IV: mutants defective in activity only. (A) Indicated mutations in AGTma were tested for effects on activity (left) or stability (right). Growth of mutants was normalized to % growth of wild-type AGTma. Average and standard error of at least three independent assays is presented. (B). Comparison of growth in activity (left) or stability (right) assay of each mutant in AGTma or AGTmi. The growth difference was obtained by normalizing growth of each mutant to either AGTma or AGTmi, as appropriate, and subtracting the difference. Mutants indicated with an asterik (**) indicate samples in which normalized growth in AGTma vs AGTmi was significantly different (p<.05).

Group IV includes eight mutants that showed near wild-type stability in AGTma and AGTmi but significantly reduced activity in AGTmi and, in all but two cases, AGTma. These mutants were previously characterized only on the minor allele (the allele they were associated with in patients) [16], and include known active site mutants G82R, H83R, W108R, and H261Q. In Fig. 4a, we characterize each mutation in the major allele. While six of these mutants have only been found to cause disease in conjunction with the minor allele, we found that that most of these mutations also show poor activity with AGTma, and thus expect these would be deleterious in patients even in the AGTma background. To identify mutants that show synergistic interaction with AGTmi, we compared the difference in activity and stability of each mutant in the major allele, with the previously reported activities and stabilities in the minor allele [16] (normalized to % of wild-type AGTmi or AGTma). Two mutations, G47R and E274D, showed significantly different (p<.05) activity in AGTma vs AGTmi, indicating interaction with the P11L/I340M mutations. In contrast to the Group II mutations that appear to interact with AGTmi to affect *stability*, these mutations appear to act with the P11L/I340M mutations to specifically affect *activity*. For example, G47R showed near wild-type stability in both AGTma and AGTmi, severely reduced activity in AGTmi [16], but retained ∼25–30% wild-type activity in AGTma. In the dimeric structure of AGT (PDB 3R9A) [25], [26], Gly47 is located between the active site loop and the N-terminal extension of the other monomer (residues 1–24). The synergic effect could be ascribed to the finding that the P11L mutation reduces the stability of the AGT dimeric structure and that the G47R substitution may affect the interaction between the two monomers [27], thus resulting in an increased population of monomeric species. E274D was found, together with R36C and S275R, to cluster together in the AGT dimer at a region distal from the active site but near P11 (Pittman et al., 2012). While molecular dynamics simulations of E274D, a conservative substitution, did not reveal any defects, simulations of the nearby S275R showed displacement of Leu211 and Ser207 that caused a partial distortion of active site loop 206–216 that is covalently bound to PLP via Lys209 [16], indicating that this region may be sensitive to perturbations that specifically affect PLP binding in the active site. While S275R and R36C show poor activity in both AGTma and AGTmi, E274D showed equal or better than wild-type activity in AGTma but severely compromised activity in AGTmi, suggesting that E274D may only affect activity when combined with P11L of the other monomer.

**Figure 5:**
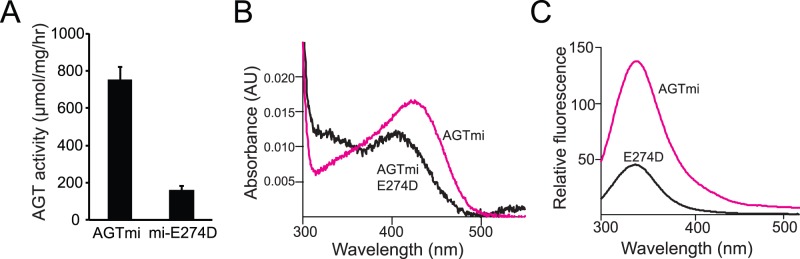
Biochemical characterization of AGTmi E274D. (**A**) Enzymatic activity of bacterially-purified AGTmi-his compared with AGTmi E274D-his. (**B**) Absorbance spectra of 10 μM AGTmi E274D or AGTmi in the presence of 100 μM PLP in 100 mM potassium phosphate buffer (pH 7.4). (**C**) Fluorescence emission spectra (excitation at 280 nm) of AGTmi and AGTmi E274D at 1 μM enzyme concentration in the presence of 100 μM PLP in 100 mM potassium phosphate buffer (pH 7.4).

To further examine the conservative E274D mutation, we expressed and purified AGTmi E274D from bacteria. Specific activity of AGTmi E274D (160 μmol/mg/hr) was significantly lower than specific activity of AGTmi (753 μmol/mg/hr) (Fig. 5A). In the presence of PLP, AGTmi E274D elutes as a dimer on size exclusion chromatography, thus suggesting that dimerization is not affected by the mutation (data not shown). We compared the intrinsic fluorescent emission spectra of AGTmi with that of AGTmiE274D in the presence of 100 μM PLP, finding that AGTmiE274D has a higher emission intensity and a 2 nm red-shifted emission maximum (Fig. 5B). This indicates that the mutation could affect the tertiary structure of the enzyme by altering the microenvironment of aromatic residues. Upon reaction (at a concentration of 10 μM) with 100 μM PLP followed by dialysis to remove unbound PLP, we found that AGTmi E274D binds approximately half the amount of PLP as AGTmi (0.95 mol PLP per mol of enzyme, compared with 2 mol PLP per mol of enzyme for AGTmi). The spectrum of the PLP-bound form of AGTmi^E274D^ shows a band at ∼410 nm, 13 nm blue-shifted when compared with that of AGTmi and characterized by a reduced absorbance, and a band at ∼320 nm, with an higher absorbance with respect to that of AGTmi (Fig. 5B). Moreover, the intrinsic fluorescence emission spectrum of the variant in the holo-form shows an about 3-fold reduced emission intensity with respect to that of holo AGTmi (Fig. 5C). Altogether, these data indicate that the E274D mutation not only affects the AGT tertiary structure, but also alters the PLP microenvironment and possibly reduces the PLP binding affinity of AGT, thus compromising the enzymatic activity. Unfortunately, we were not able to determine the equilibrium constant for the formation of the apoE274D-PLP complex. In fact, when we depleted PLP from purified AGTmi-E274D preparations, we found that apoE274D is prone to a strong aggregation process that resulted in protein precipitation on filters and prevented *in vitro* spectroscopic studies. Results with the E274D variant are consistent with previous molecular dynamics studies on S275R [16] and biochemical and structural studies of the S187F variant [28], which suggest that mutations within residues that do not directly interact with the coenzyme may still result in a local change in active site structure and PLP binding.

## Discussion

Here, we undertook a systematic characterization of activity and stability of 34 mutations in alanine: glyoxylate aminostransferase associated with primary hyperoxaluria type I, using two yeast-based assays. Of these 34 mutants, 18 were completely uncharacterized, and only a few had been analyzed for effects on stability. While half of these mutants had been previously characterized for effects on activity in AGTma or AGTmi (and some in both alleles), these experiments were often carried out by different groups using different approaches, often yielding quite different results, and only for seven mutants had both alleles been analyzed. This study, characterizing over 30 mutations in two polymorphic alleles side-by-side in the same two assays, provides the largest systematic comparison of PH1 associated mutations in AGT to date.

Interestingly, when we analyzed each mutant in the yeast activity assay in the reported polymorphic background (AGTma or AGTmi) in which it was found, we found very few intermediate phenotypes. Mutant activity, based on growth, was either similar to wild-type (in the case of Group III mutants) or it was below 20% of wild-type (AGTma or AGTmi, as appropriate). In previous studies using the yeast complementation assay [7], the only two mutants to show intermediate phenotypes were AGTmi-G170R and AGTmi-F152I (between 30–50% wild-type activity), both of which are mistrafficking mutants. The fact that the disease variants (excluding the mistrafficking variants) show severe, rather than moderate activity phenotypes in the complementation assay suggests that even significant reductions (e.g. 30% of wild-type) in AGT enzymatic activity may not be disease-causing. As such, rescue of destabilized variants by pharmacological agents that improve enzymatic yield even a small amount could greatly improve disease outcome.

We characterized five reported disease variants (D129H, L153V, I279M, Q282R, and S287T) that had relatively little discernable effect on activity or stability in our yeast model. We examined four of these (all but I279M) for peroxisomal trafficking defects in mammalian cells, and for differences in total protein levels as visualized by western blotting, but did not observe any defects. It is possible that these mutations may affect activity in ways that we have not examined. For example, we did not specifically examine protein stability or activity within the peroxisome (a mutation such as I279M could be affected by methionine oxidation in the oxidizing environment of the peroxisome), or activity may be particularly susceptible to cofactor (PLP) levels that may be different in the mammalian cell peroxisome.

We identified 12 mutations that affect growth and/or stability on the minor allele (AGTmi) to a greater degree than on the major allele (AGTma). Of these, four mutations (G161C, G161S, L166P, and C253R) were previously shown to have a greater effect on AGTmi activity [24], and our studies confirm these prior results. We identified eight new mutations (G47R, I56N, E95K, M195L, D243H, E274D, P319L, A368T) that also synergize with AGTmi, six of which appear to have stability defects. These mutations can thus be included with other mutations known to synergize with the AGTmi allele, which include I244T, G170R, F152I, R233C, and R233H [6], [24]. While it is possible that some of these new variants could be candidates for mitochondrial mistrafficking, as is seen with variants AGTmiG170R and AGTmiF152I [6], [9], they show a very different phenotype from these mistrafficking mutants. In the same assay, AGTmiG170R and AGTmiF152I showed 30–50% wild-type activity when compared with AGTmi [7], while all tested mutants here showed <20% activity (and generally, <10% activity).

**Figure 6:**
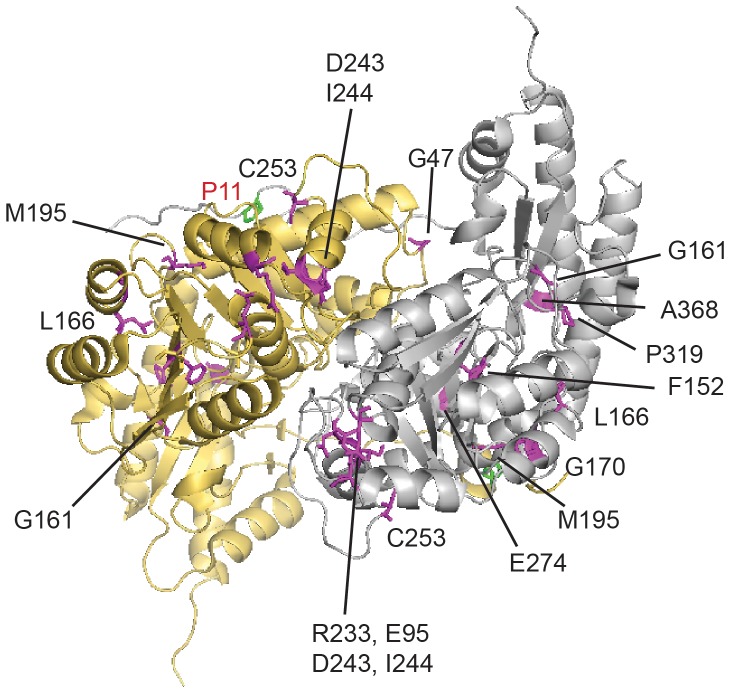
Structure of AGT dimer showing mutations synergistic with minor allele variant. P11L residue associated with AGTmi is shown in green, disease mutations are shown in magenta.

When we mapped all 12 mutations that were found to interact with AGTmi on the dimeric structure (PDB 3R9A), we find they are localized throughout the protein sequence (Fig. 6). With the exception of E274D, they are not located near the P11L and I340M residues associated with AGTmi on either monomer of the dimer. Several of the residues interacting with AGTmi are clustered, including Arg233 and Glu95 (separated by 3 Å); Gly170, Leu166, and Met195 (4–6 Å apart); and Asp243, Ile244, and Cys253 (7–8 Å apart). Some of the mutations in these clusters are localized at or near to the dimer interface and may thus destabilize the dimeric structure. In general, these perturbations are far enough from P11L that they would not be expected to interact directly with this substitution, but together the changes may synergistically influence stability more globally or indirectly.

Intriguingly, we observed significant differences in interaction with AGTma vs AGTmi for several variants that had mutations at the same site. Gly161 has been found mutated to Arg, Cys, or Ser in different patients, where G161R was found in a patient in conjunction with AGTma, while G161C and G161S were found in conjunction with AGTmi. In agreement with the patient genotypes, we found G161R to be most severe, significantly reducing stability and activity in both AGTma and AGTmi. Gly161 is located in a loop that also contains active site residue Ser158, and substitution to the bulkier, charged Arg does not appear to be tolerated for either activity or stability. In contrast, Cys and Ser are more conservative substitutions, and the G161C and G161S phenotypes were milder, retaining over 50% activity in AGTma, but poor activity and stability in AGTmi. These activity results are consistent with previous measurements of G161S and G161C in AGTma and AGTmi backgrounds [24]. The stability results are in agreement with studies of Gly161 mutant variants expressed in CHO cells, which found the soluble fraction of extracts contained less than 10% of AGTmi levels for all three Gly161 variants [18]. Gly161 is located near Gly170, Leu166, and Pro319, three other regions affected by AGTmi, and this region appears to be particularly sensitive to AGTmi changes. A second set of mutations with differing severity also in this region were M195R and M195L, located on an adjacent active site loop close to Gly170. These mutations were both found associated with AGTmi in patients. While M195L showed a deleterious phenotype only in AGTmi, M195R, the more severe mutation, showed <1% activity and stability in AGTma as well (Fig. 1).

Most of the Group II mutants showing differential effects in AGTma vs AGTmi were identified in patients in association with AGTmi. One exception to this was I56N, which was identified in both AGTma and AGTmi, suggesting this mutation is likely deleterious in AGTma. Our studies showed nearly full activity of I56N in AGTmi, but poor (<5%) growth in the stability assay, confirming that this variant does not show wild-type behavior in AGTma. For two mutants identified in Group II, D243H and P319L, the associated allele was not reported, however our results would suggest these mutations are likely associated with AGTmi.

With several previously characterized mutations in Group II, we observed differences between *in vitro* activity measurements and our *in vivo* activity determinations. Previous activity determination of AGTmi G161S and G161C in the purified form had revealed relative activities of ∼60% and ∼30% compared with AGTmi [19]. In contrast, our yeast cell based activity assay showed these mutants to have <5% AGTmi activity. These different results likely reflect differences between *in vitro* measurements using purified protein and the cellular assay, which reflects a combination of activity and stability, and is affected by aggregation and degradation of mutant proteins [16].

Finally, we carried out a detailed characterization of the E274D variant. This variant contains a conservative substitution at a site that appears to be particularly sensitive to perturbation in the context of the AGTmi allele, but not in the context of the AGTma allele. In the dimeric structure of AGT (Fig. 6), this mutation is located near P11L of the opposing subunit, raising the possibility that a heterodimer formed by AGTmi-E274D and AGTma would have normal activity. While many of the other mutations that cause disease in combination with AGTmi act by decreasing protein stability, E274D appears to act through a different mechanism, specifically affecting activity. The spectral properties of the variant in the purified form indicate that the mutation affects both holoAGTmi tertiary structure and PLP binding, thus possibly explaining why the variant displays a reduced specific activity.
